# APP-CD74 axis mediates endothelial cell-macrophage communication to promote kidney injury and fibrosis

**DOI:** 10.3389/fphar.2024.1437113

**Published:** 2024-09-16

**Authors:** Bin Liu, Faping Li, Yuxiong Wang, Xin Gao, Yunkuo Li, Yishu Wang, Honglan Zhou

**Affiliations:** ^1^ Department of Urology II, The First Hospital of Jilin University, Changchun, Jilin, China; ^2^ Key Laboratory of Pathobiology, Ministry of Education, Jilin University, Changchun, Jilin, China

**Keywords:** renal fibrosis, single-cell RNA sequencing, endothelial cell, macrophage, intercellular communication

## Abstract

**Background:**

Kidney injuries often carry a grim prognosis, marked by fibrosis development, renal function loss, and macrophage involvement. Despite extensive research on macrophage polarization and its effects on other cells, like fibroblasts, limited attention has been paid to the influence of non-immune cells on macrophages. This study aims to address this gap by shedding light on the intricate dynamics and diversity of macrophages during renal injury and repair.

**Methods:**

During the initial research phase, the complexity of intercellular communication in the context of kidney injury was revealed using a publicly available single-cell RNA sequencing library of the unilateral ureteral obstruction (UUO) model. Subsequently, we confirmed our findings using an independent dataset from a renal ischemia-reperfusion injury (IRI) model. We treated two different types of endothelial cells with TGF-β and co-cultured their supernatants with macrophages, establishing an endothelial cell and macrophage co-culture system. We also established a UUO and an IRI mouse model. Western blot analysis, flow cytometry, immunohistochemistry and immunofluorescence staining were used to validate our results at multiple levels.

**Results:**

Our analysis revealed significant changes in the heterogeneity of macrophage subsets during both injury processes. Amyloid β precursor protein (APP)-CD74 axis mediated endothelial-macrophage intercellular communication plays a dominant role. In the *in vitro* co-culture system, TGF-β triggers endothelial APP expression, which subsequently enhances CD74 expression in macrophages. Flow cytometry corroborated these findings. Additionally, APP and CD74 expression were significantly increased in the UUO and IRI mouse models. Immunofluorescence techniques demonstrated the co-localization of F4/80 and CD74 *in vivo*.

**Conclusion:**

Our study unravels a compelling molecular mechanism, elucidating how endothelium-mediated regulation shapes macrophage function during renal repair. The identified APP-CD74 signaling axis emerges as a promising target for optimizing renal recovery post-injury and preventing the progression of chronic kidney disease.

## 1 Introduction

The estimated global incidence of chronic kidney disease (CKD) is approximately 8–16% and has increased recently as a public health problem (Jha et al., 2013). Fibrosis is a hallmark and driver of CKD, leading to progressive functional impairment (Rojas-Morales et al., 2019). Renal fibrosis is caused by excessive inflammation and maladaptive repair. Therefore, it is necessary to clarify the potential pathophysiological changes in kidney fibrosis and seek effective interventions to delay CKD progression.

Kidney fibrosis involves multiple cytokine and cell types. Fibroblasts differentiate into α-SMA^+^ myofibroblasts, leading to excessive deposition of the extracellular matrix (ECM) (Kuppe et al., 2021). Moreover, tubular epithelial cells are actively involved in fibrosis through epithelial-mesenchymal transformation (EMT) (Kriegel et al., 2010), complement activation (Boor et al., 2007), autophagy (Li et al., 2016a), apoptosis (Huang et al., 2019), and lipid nephrotoxicity (Miguel et al., 2021). Macrophage polarization plays an opposite role in renal fibrosis owing to its anti-inflammatory or pro-inflammatory properties (Lu et al., 2018). The phagocytosis of macrophages has been extensively studied in the context of lung and liver injury repair and fibrosis (Wuyts et al., 2013; Li et al., 2016b; Cheng et al., 2021).

Furthermore, various types of kidney cells interact in a coordinated and regulated manner to promote injury repair. The development of high-throughput single-cell RNA sequencing (scRNA-seq) technology has facilitated the simultaneous detection of gene expression levels in thousands of cells at the resolution of a single cell. Analysis of cell subpopulation structure and development trajectory is crucial for understanding cell heterogeneity during disease progression. The scRNA-seq technology has been reported to facilitate exploring the heterogeneity of macrophages in renal injury (Conway et al., 2020), cancer (Koh et al., 2021), and transplant (Malone, 2021); however, most studies have focused on the interaction between macrophages and other immune cells. For non-immune cells, some studies have documented that macrophages regulate angiogenesis, participate in inflammation-related vascular diseases (such as atherosclerosis, inflammatory bowel disease, and tumors), mediate programmed cell death, promote phenotypic transformation (such as epithelial-mesenchymal transition, endothelial-mesenchymal transition, and pericyte-myofibroblast transdifferentiation), and inhibit tubular epithelial cell cycle arrest (Kalucka et al., 2017; Deng et al., 2022). However, the influence of endothelial/epithelial cells on macrophages mainly manifests in the recruitment of macrophages by endothelial cells and the induction of macrophage polarization to M1 or M2 phenotypes by tubular epithelial cells during different stages of acute kidney injury (AKI), thus affecting their functional roles (Kalucka et al., 2017; Deng et al., 2022). These studies can partially explain the mechanisms of fibrosis (as inflammation is associated with fibrosis progression), but few studies have reported the direct pro-fibrotic effects of epithelial/endothelial cells through macrophages. Moreover, the specific mechanisms and signaling pathways by which epithelial/endothelial cells influence macrophage behavior and function remain unclear.

Conway et al. (2020) established a reversible unilateral ureteral obstruction (RUUO) mouse model to simulate kidney injury and fibrosis regression. Mice were subjected to unilateral ureteral obstruction (UUO) for 7 days before RUUO treatment. Mice were divided into control (sham), UUO-treated 2 days (UUO2), UUO-treated 7 days (UUO7), and RUUO-treated 2-week groups according to the modeling time. Plate- and droplet-based single-cell sequencing of the obtained kidneys was performed. They demonstrated the heterogeneity of myeloid cells and discovered a novel subpopulation of macrophages, characterized by the expression of Mmp12, that contributes to the process of kidney injury repair. They suggested that Mmp12^+^ macrophages represent a therapeutic target for inhibiting renal disease progression or promoting its regression.

In this study, we utilized the scRNA-seq dataset of Conway et al. (GSE140023) from the Gene Expression Omnibus (GEO) database and shifted the focus of the study from myeloid heterogeneity to its transformation process and communication with other cells, particularly non-immune cells. We identified a strongly correlated set of pro-fibrotic ligand-receptor (L-R) pairs between endothelial cells (ECs) and macrophages using “CellChat,” a newly developed tool for quantitative inference and analysis of intercellular communication (Jin et al., 2021). Furthermore, we validated these results using the scRNA-seq transcriptome dataset of the mouse ischemia-reperfusion injury (IRI) model published by (GSE161201) Ide et al. (2021). Our data show extensive alterations in communication between non-immune cells and macrophages during renal injury, implicating the importance of cell communication in kidney fibrosis. Blocking this change in intercellular communication may play an important role in anti-fibrotic therapies.

## 2 Materials and methods

### 2.1 Data correction and quality control

Raw scRNA-seq profiling GSE140023 of the RUUO mouse model was obtained from the GEO database (https://www.ncbi.nlm.nih.gov/geo/). Quality control assessments were performed to remove potentially empty droplets and doublets. For all four groups, cells with fewer than 300 genes and genes expressed in fewer than three cells were removed. Subsequently, the “FindIntegrationAnchors” function recommended by the Stuart et al. (2019) was used to eliminate batch effects. In total, 17,934 genes were identified. We retained 3,926, 2,095, 4,261, and 3,083 cells from the sham, UUO2, UUO7, and RUUO groups, respectively. The remaining data were used to produce a combined dataset. The independent external IRI dataset (GSE161201) used for validation was also obtained from the GEO database and separately extracted scRNA-seq data for day 7. After using the Seurat algorithm for standardization, we obtained 19,841 transcriptome data from 6,564 cells for subsequent analysis.

### 2.2 Dimensionality reduction, visualization, and cell cluster identification

After quality control, the Seurat R package v.3.06 was used to analyze the data for dimensionality reduction and cell clustering (Stuart et al., 2019). UMAP with a resolution of 0.5 and a dimension of 30 was used to visualize the main cell clusters of the integrated dataset. Relatively overexpressed genes in a cluster were identified as marker genes for the population compared with all other cells. Using the “FindAllMarkers” function in Seurat, which followed the nonparametric Wilcoxon rank-sum test, differential gene testing was performed to obtain the top markers for each cluster. An “avg_log2FC” (average log fold change) and an “adj_Pval” (Bonferroni-adjusted *P* values) for each gene were returned. We ranked the genes in the order of avg_log2FC and visualized them using heatmaps. Cluster-specific markers were retained with log2FC > 0.25. Afterward, marker genes were used to annotate each cell cluster and visualized using dot or violin plots. The procedures for clustering the myeloid cells and UUO7 group were as described above.

### 2.3 Functional enrichment analysis

Online Venny 2.1 software (https://bioinfogp.cnb.csic.es/tools/venny/) was used to construct Venn diagrams and screens for differentially-expressed genes (DEGs). Functional enrichment analysis was performed on DEGs using Metascape (https://metascape.org/gp/index.html#/main/step1) (Zhou et al., 2019). The KEGG and GO enrichment DEG analyses were performed using the DAVID (version 6.8) online tool (https://david.ncifcrf.gov/).

### 2.4 Pseudo-time trajectory analysis

First, we extracted myeloid cell clusters from the Seurat object. Pseudo-time was performed using the Monocle 2 (version 2.18.0) algorithm with default parameters and scaled from 0 to 1 (Qiu et al., 2017). The indicated channels were used as input dimensions. Subsequently, the “differentialGeneTest” function in the Monocle 2 package was used to recognize the hub genes in each cluster. The first 400 genes were screened as sequencing genes according to q values (q < 0.01). In addition, the “reduce dimension” function reduced expression profiles to 2 dimensions via the DDRTree method, with max_components = 2. The “orderCells” function arranged the myeloid cells in a specific order and assigned a “pseudo-time” value. Pseudo-time-dependent genes were determined via the “differentialGeneTest” function. Thus, we ordered the cells in a pseudo-time trajectory.

### 2.5 Cell-cell communications

The CellChat R package (version 1.0.0) was used to infer and quantify cell-cell communication interactions involved in cell clusters (Jin et al., 2021). The information flow of each signaling pathway was calculated and compared, defined as the inference of all communication probabilities between all cell clusters in the network. The “netAnalysis_signalingRole_heatmap” function of CellChat was used to recognize outgoing and incoming signals of cell types, which were visualized through the “netVisual_aggregate” function.

### 2.6 Cell culture

All cell lines were purchased from Haixing biosciences. Human brain microvascular endothelial cells (BMEC) and human umbilical vein endothelial cells (HUVEC) were cultured in DMEM (Meiunbio, MA0212) medium containing 10% (vol/vol) fetal bovine serum (Biological Industries, 04-001-1ACS) and 1% penicillin-streptomycin solution (Biosharp, BL505A). Human monocytic-leukemia cells (THP-1) were cultured in 1,640 medium containing 10% (vol/vol) heat-inactivated fetal bovine serum, 0.05 mM β-mercaptoethanol (Aladdin, 60-24-2) and 1% penicillin-streptomycin solution. THP-1 cells were treated with 100 ng/mL Phorbol-12-myristate-13-acetate (Beyotime, S1819) to induce differentiation to macrophages for subsequent experiments.

### 2.7 RNA isolation and quantitative RT-PCR

The total RNA was isolated from cultured cells using Trizol reagent (Ambin, 411502). Hifair III 1st strand cDNA Synthesis SuperMix (Yeasen, H8223900) was used to synthesize cDNA from 1 μg of total RNA following the manufacturer’s instructions. In addition, quantitative RT-PCR was conducted with diluted cDNA, gene-specific primers, and Hieff qPCR SYBR Green Master Mix (Yeasen, H2306210). Temperature and time were set according to the manufacturer’s instructions. We calculated the relative levels of target gene mRNA using the 2^−ΔΔCT^ method.

Primers used in this study: APP (forward AAC​CCC​AGA​TTG​CCA​TGT​TCT, reverse GCA​GTT​CAG​GGT​AGA​CTT​CTT​GG), CD74 (forward GGC​AAC​ATG​ACA​GAG​GAC​CA, reverse GCT​CTC​ACA​TGG​GGA​CTG​G), GAPDH (forward AAG​GGT​CAT​CAT​CTC​TGC​CC, reverse CAT​GGA​CTG​TGG​TCA​TGA​GT).

### 2.8 Protein extraction and western blotting

Cells were harvested and homogenized on ice using RIPA lysis buffer (Beyotime, P0013C) containing 1:100 protein phosphatase inhibitor (Solarbio, P1260), and 1:100 PMSF solution (Beyotime, ST507). After centrifugation at 12,000 rpm for 20 min at 4°C, the supernatant concentrations were determined by BCA assay (Solarbio, PC0020) according to its instructions. Equal amounts of protein samples were boiled in 5*SDS-PAGE protein loading buffer (Yeasen, S3301100) for 5 min, and subjected to 8%–12% SDS-PAGE gels (self-configuring) and then transferred to PVDF membranes (Immobilon, R1PB81493).

The membranes were subjected to a number of steps such as sealing with 5% skimmed milk powder, washing with TBS-T, incubating with primary antibodies and coupling with secondary antibodies. Subsequently, ECL chemiluminescent reagent (Meilunbio, MA0186-1) was used to visualize the band densities of target genes and recorded using a CCD camera (Tanon-4600).

Antibodies used in the present study: anti-GAPDH (Proteintech, 10494-1-AP), anti-beta-Tubulin (Proteintech, 10094-1-AP), anti-APP (PTM-biolab, PTM-20007) and anti-CD74 (PTM-biolab, PTM-6631).

### 2.9 Co-immunoprecipitation (Co-IP)

Use the Co-IP Pierce™ kit (ThermomFisher, United States, Stock No. 88804) following the manufacturer’s instructions. All procedures were performed on ice. The 1,000 ug of protein was diluted to 500 ul by mixing with 5 ug of APP antibody and incubated overnight at 4°C; 25 µL (0.25 mg) of Pierce Protein A/G beads were then added, and protein-antibody complex supernatants were obtained by manual immunoprecipitation followed by elution, and protein blotting analyses were performed on these samples.

### 2.10 UUO and IRI mouse model establishment

Eight-week-old male BALB/c mice, weighing approximately 20 ± 3 g, were obtained from Shanghai Model Organisms Center, Inc. (Shanghai, China). They were accommodated in the animal barrier facility at Jilin University under a 12-h light/dark cycle, with free access to food and water.

To simulate the process of kidney injury, we established the UUO as well as the IRI model following the previous methods (Conway et al., 2020; Ide et al., 2021). Three groups were set up: sham-operated group (n = 5), UUO group (n = 5), and IRI group (n = 5). Mice were euthanized 7 days after UUO and IRI surgery to obtain kidney tissue. The collected kidneys were stored at −80°C for subsequent western blot detection or fixed in formalin for 1 week for preparation of paraffin embedding and sectioning for subsequent histological examination.

The animal study was approved by institutional animal care and use committee of Jilin University (approval number 419, 2023). The study was conducted in accordance with the local legislation and institutional requirements.

### 2.11 Hematoxylin and eosin (HE) staining and injury score

For HE staining, a standard protocol was followed, encompassing the following steps: dewaxing, dehydration, hematoxylin staining, differentiation, bluing, eosin staining, further dehydration, clearing, and coverslips.

The Injury Score consists of the Tubular Injury Score, the Interstitial Fibrosis Score and the Inflammatory Cell Infiltration Score. Each item is scored 0–3 depending on the degree of injury. The average of the three scores gives the injury score.

### 2.12 Masson staining

For Masson staining, we prepared 3-μm-thick paraffin sections. The Masson staining kit was purchased from Solarbio (Stock No. G1346). The Masson staining followed the kit’s standard protocol, including: dewaxing, hydration, Weigert’s iron hematoxylin, Biebrich scarlet acidic magenta, phosphomolybdenum phosphotungstic acid, aniline blue, acetic acid, dehydration, clearing and coverslips. ImageJ software was used to perform semiquantitative analysis.

### 2.13 Immunohistochemistry

The kidneys of mice were obtained by formalin fixation and then paraffin embedded for sectioning. The sections were then dewaxed with xylene and rehydrated with a gradient concentration of ethanol. In the next step, the immunohistochemistry kit (Maxim Biotechnology, KIT-0100M) was used as directed by its manufacturer. Briefly, sections were subjected to high temperature repair in citrate buffer, closured with a blocking solution, first incubated with primary antibodies at 4°C overnight, then with biotin-coupled secondary antibodies on the second day, and finally with streptavidin-HRP. Target protein expression was observed using DAB (Maxim biotechnology, DAB-1031) as a chromogenic agent and hematoxylin as a re-staining agent. ImageJ software was used to perform semiquantitative analysis.

### 2.14 Immunofluorescence

Dewaxing and antigen repair of the sections were performed in the same way as the immunohistochemical processing. After blocking with 5% BSA, F4/80 (Invitrogen, 14-4801-82) and CD74 antibody were diluted at 1:100 and incubated overnight at 4°C, followed by incubation with fluorescently labeled secondary antibody. Finally, the nuclei were stained with DAPI and then photographed.

### 2.15 Flow cytometry

The treated cells were collected and incubated with CD74 primary antibody at a dilution of 1:50, after which secondary antibody with fluorescent labels was incubated for staining, followed by washing with PBS and centrifugation after each operation. All operations were carried out on ice and protected from light. Ultimately, analysis was carried out using an LSR Fortessa X-20 cell analyzer (BD Biosciences). All file outputs were in FCS format and analyzed using FlowJo software version 10.

### 2.16 Statistical analysis

Statistical analysis was conducted using GraphPad Prism 9. Data were expressed as mean ± SD. Differences between two groups were tested using a t-test (unpaired, two-tailed) and there were at least three independent experiments in each group. And *P* < 0.05 was regarded as a statistically significant difference.

## 3 Results

### 3.1 Identifying renal cell types in UUO mice

**FIGURE 1:**
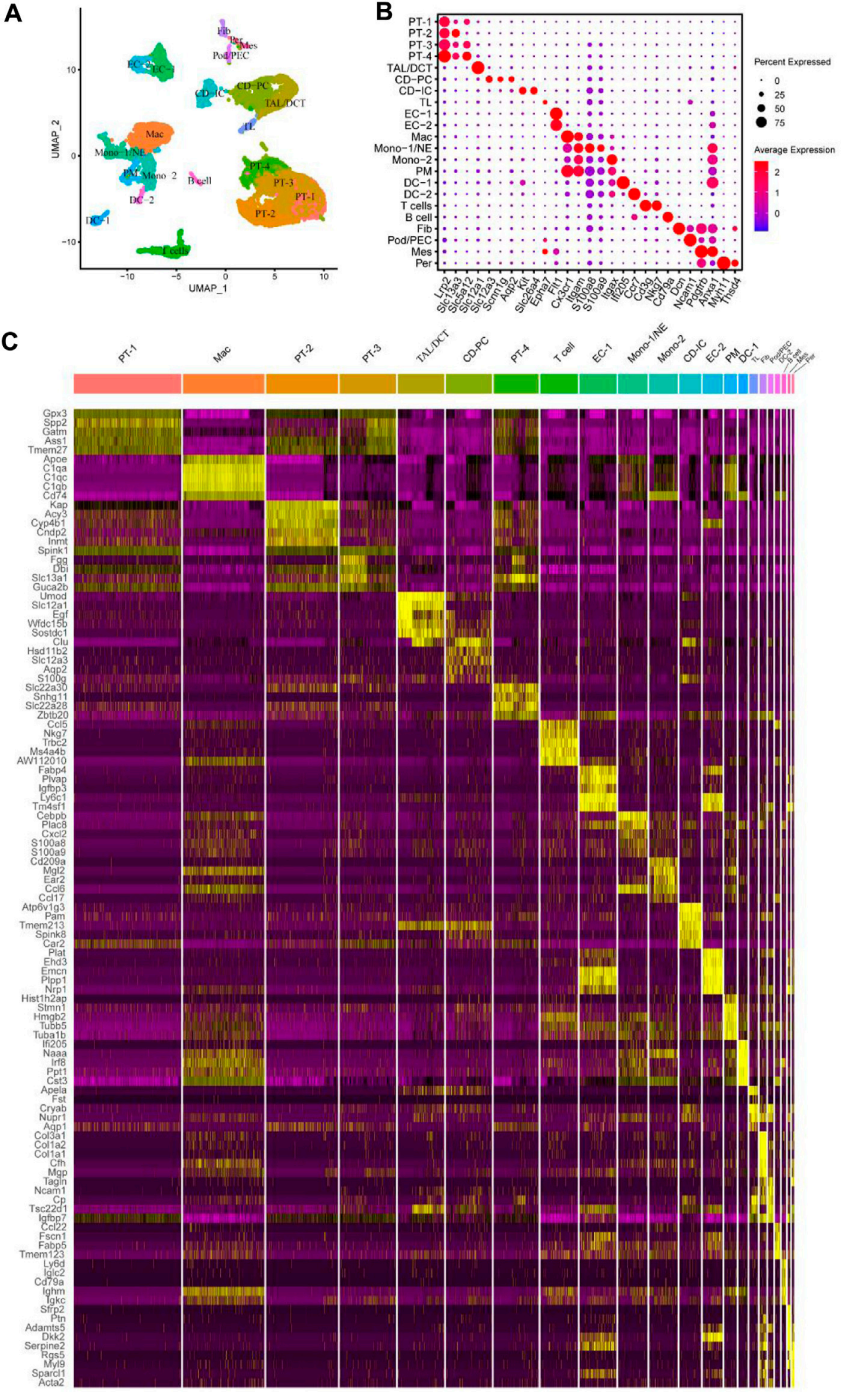
Identified different cell subsets of mouse UUO kidneys using the online published single-cell RNA sequencing (scRNA-seq) datasets. **(A)** Identified and visualized (0–21) clusters, including all kidney cell populations of different time points (sham, UUO-2, UUO-7, and R-UUO). **(B)** Dot plot showing differential expression levels of some well-known marker genes for cell clusters. **(C)** Heat map displaying gene expression patterns of the top five in each cluster. UUO, unilateral ureteric obstruction; R-UUO, reversible-UUO; CD-IC, collecting duct intercalated cells; CD-PC, collecting duct principal cells; DC, dendritic cells; DCT, distal convoluted tubule; EC, endothelial cells; Fib, fibroblasts; Mes, mesangial cells; Mac, macrophages; Mono, monocyte; NE, neutrophil; PEC, parietal epithelial cells; Per, pericytes; PM, proliferating monocyte; Pod, podocytes; PT, proximal tubule; TAL, thick ascending limb; TL, thin limb cells.

We applied quality control to the scRNA-seq results mentioned in the “Materials and Methods” and obtained 17,934 genes in 13,365 cells in all four sample groups (GSE140023). As an organ responsible for filtering metabolic waste from the body, maintaining the water and salt balance, and secretory functions, the kidney has a complex cellular composition. After clustering and visualization, the cells were divided into 22 clusters (Figure 1A). Clusters were manually annotated based on the top five highly-expressed genes referring to previous relevant literature (Kirita et al., 2020; Ide et al., 2021) and the Cellmarker database (http://xteam.xbio.top/CellMarker/index.jsp) (Zhang et al., 2019) (Figures 1B, C; Supplementary Spreadsheet S1). This is consistent with the clustering results of Conway et al. (2020). We further subdivided some cells specific to glomeruli and cell subsets.

The epithelial cells in each segment of the nephron showed different gene expression patterns because of their distinct secretion and reabsorption functions. Notably, proximal tubule (PT) cells in the lower right corner of uniform manifold approximation and projection (UMAP) had the largest number; they were subdivided into PT-1‒4 based on their DEGs. DEGs between the four clusters were identified using Venny online software (Supplementary Figure S1A); functional enrichment analysis was performed using Metascape (Supplementary Figures S1B–E). The four PT cell subsets have different biological functions, including the citric acid cycle and respiratory electron transport, fatty acid metabolic process, post-translational protein phosphorylation, and mRNA processing. In this study, other epithelial cells, including thick ascending limb/distal convoluted tubules (TAL/DCTs), collecting duct principal cells (CD-PCs), collecting duct intercalated cells (CD-ICs), and thin limb cells (TLs) were also detected. The number of some cells specific to glomeruli, such as podocytes (Pods), parietal epithelial cells (PECs), mesangial cells (Mes), and pericytes (Pers), was low, which may have been related to the difficulty of glomerular cell isolation. Macrophages also accounted for a large proportion; other immune cells were clustered around and comprised T cells, B cells, monocytes (Monos), neutrophils (NEs), proliferating monocytes (PMs), and dendritic cells (DCs).

### 3.2 Heterogeneity of macrophages in renal injury and repairment

**FIGURE 2:**
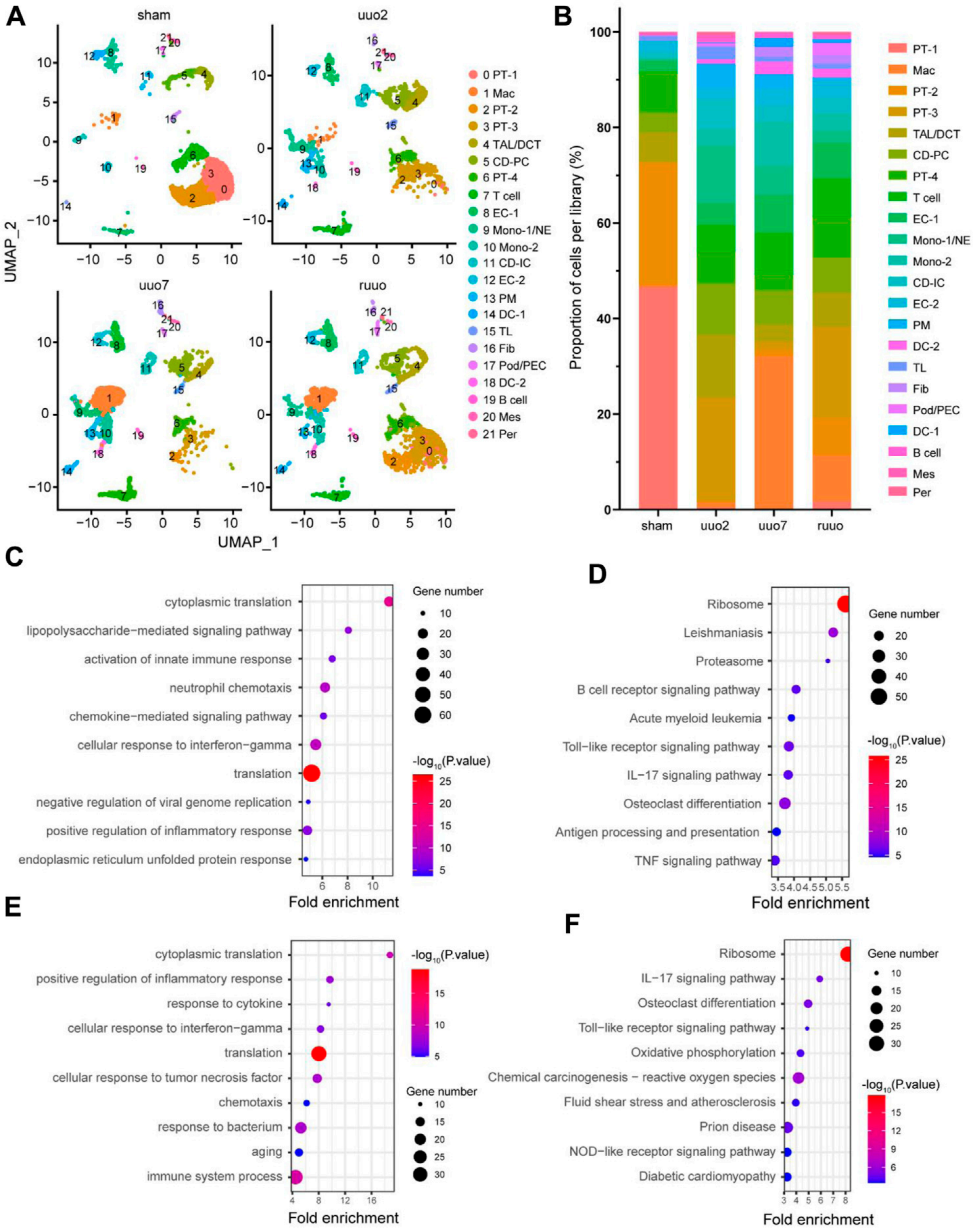
Heterogeneity of cell populations. **(A)** UMAP plot of sham, UUO-2, UUO-7, and R-UUO groups. **(B)** The changes in relative proportions of each cell type by time point. GO **(C)** and KEGG **(D)** functional enrichment analyses of differentially expressed genes in macrophages cluster between UUO7 and sham. GO **(E)** and KEGG **(F)** functional enrichment analyses of differentially expressed genes in macrophages cluster between R-UUO and UUO7. UUO, unilateral ureteric obstruction; R-UUO, reversible-UUO; GO, gene ontology; KEGG, kyoto encyclopedia of genes and genomes.

During the process of UUO (sham surgery, UUO2, UUO7, and RUUO), a number of renal cells were likely functionally affected. To clarify these effects, we re-clustered the cell populations of each group according to the same criteria (Figure 2A). The details of each type of cell number and percentage are shown in Figure 2B and Supplementary Table S1. As expected, dramatic changes were observed. The proportion of macrophages increased 51-fold, from 0.63% at the beginning to 32.26% at UUO7, and decreased to 9.79% after RUUO. EC-1 initially increased from 2.25% to 7.92%, then decreased slightly to 7.52% after RUUO. A similar trend was observed for EC-2. The findings in epithelial cells, where PT-1, PT-2, and PT-4 were significantly reduced after UUO exposure, were dominant in the sham group, while PT-3 was significantly increased. Such drastic changes are related to the injury and death of epithelial cells during UUO injury and their dedifferentiation and apoptosis (Cachat et al., 2003; Chevalier et al., 2009).

We hypothesized that such acute changes in macrophages are actively involved in kidney damage and repair. Functional enrichment analyses of DEGs between different time points were performed to determine what happens to macrophages during that time. Gene ontology (GO) biological process analysis revealed that compared with the sham period, UUO7 macrophages mainly affected the activation of the innate immune response, positive regulation of the inflammatory response, and neutrophil chemotaxis. (Figure 2C). The Kyoto Encyclopedia of Genes and Genomes (KEGG) pathway was mainly enriched in the B-cell receptor signaling pathway, antigen processing and presentation, osteoclast differentiation, and proteasome pathways (Figure 2D). These results are consistent with those of previous studies (Hamidzadeh et al., 2017; Muntjewerff et al., 2020; Ross et al., 2021). After relieving ureteral obstruction through ureteral re-anastomosis, macrophages focused more on cytoplasmic translation, ribosomal pathway, and osteoclast differentiation (RUUO vs. UUO7), (Figures 2E, F).

### 3.3 Pseudo-time distribution of macrophages

**FIGURE 3:**
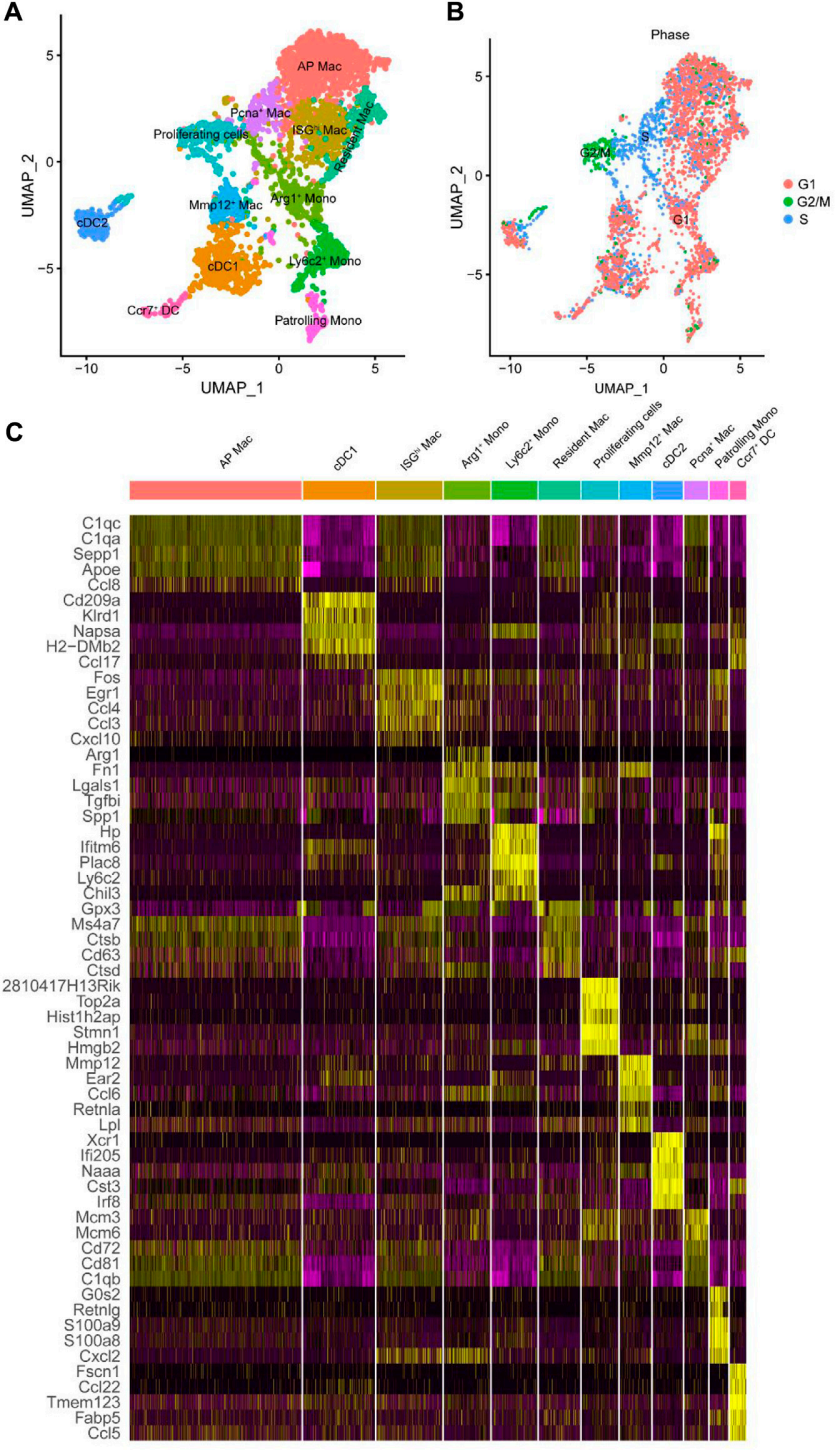
Further subdivided myeloid cell subtypes. **(A)** UMAP plot of the identified myeloid cell subtypes. **(B)** UMAP plot of the cell cycle of myeloid cell subtype. **(C)** Heat map displaying gene expression patterns of the top five in each cluster. Mac, macrophages; Mono, monocyte; AP, antigen presentation; ISG^hi^, interferon-stimulated genes high expression; Pcna, proliferating cell nuclear antigen; Arg1, arginase-1; Mmp12, matrix metalloproteinase 12; Ly6c2, lymphocyte antigen 6 C2; Ccr7, C-C motif chemokine receptor 7; DC, dendritic cells; cDC, conventional DC.

These results suggested that macrophages have different biological functions at different stages. Macrophages can be differentiated into distinct phenotypes based on the tissue microenvironment. Because macrophages are derived from myeloid cells, we screened myeloid cells previously identified in Figure 1A for detailed subclassification. They were subdivided into 12 groups based on highly expressed (Figure 3C) and marker genes (Supplementary Figure S2; Supplementary Spreadsheet S2). The detailed cell types were mapped directly onto the UMAP plot (Figure 3A). Consistent with Conway et al. (2020), we maintained the same classification of DC, monocytes, and proliferating cells. Monocytes were divided into Arg1+, Ly6c2+, and patrolling monocytes; DC was divided into the conventional cDC1, cDC2, and Ccr7+ clusters. There was a group of proliferating cells with high expression of PCNA and Mki67. Mki67 represents cells in a proliferative state. It is only expressed in the nucleus of G1/S/G2 and mitotic stages but not in the G0 stage (Wu et al., 2021). Classification by the cell cycle using the Seurat package also confirmed that these proliferating cells were in the G2/M phase (Figure 3B). In addition, macrophages were divided into five clusters, including Mmp12+ and resident macrophages, consistent with the original study. The other three clusters were named antigen presentation (AP) macrophages, interferon-stimulated gene high expression (ISGhi) macrophages, and proliferating cell nuclear antigen (Pcna+) macrophages. The enriched genes of AP macrophages, such as Ctss, Cd81, and B2m, were shown to play a role in antigen presentation. ISGhi macrophages showed high expression of CCL4, TNF, and other interferon-related genes. Pcna+ myeloid clusters, likely to be proliferating macrophages, were highly expressed in Pcna, but did not express Mki67.

**FIGURE 4:**
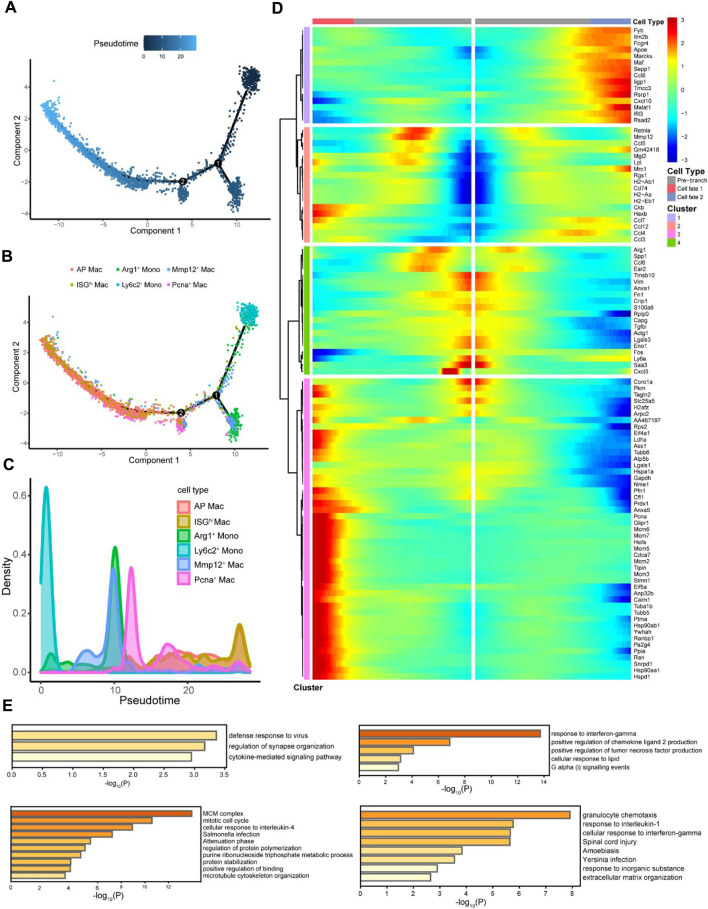
Pseudo-time analysis explored the differentiation process of macrophages. **(A)** Cells were colored based on pseudo-time. **(B)** Distribution of myeloid cell subtypes on the pseudo-time trajectory. **(C)** Density of myeloid cell subtypes changed with pseudo-time trajectory. **(D)** Starting from point 2 **(B)**, monocytes with two fates exhibit four different gene expression clusters. **(E)** Functional enrichment analyses of different gene expression clusters were expressed as the -log10 (P) adjusted for multiple comparisons.

To explore the differentiation trajectory from monocytes to macrophages, subtypes of monocyte and macrophage clusters subdivided in Figure 3A were distributed according to the pseudo-time trajectory (Figures 4A, B), showing dynamic changes (Figure 4C). Ly6c is a member of the GPI-anchored cell surface glycoprotein polygene family containing two homologous components, Ly6c1 and Ly6c2, and is expressed in several immune cell types (Spindler et al., 2010; Hanninen et al., 2011; Omi et al., 2014). Monocytes expressing Ly6c are considered progenitors of inflammatory macrophages (Shi and Pamer, 2011). The number of Ly6c2+ monocytes was dominant at the beginning; there were two obvious time nodes for differentiation into Arg1+ monocytes, Mmp12+, Pcna+, AP, and ISGhi macrophages, consistent with the results of Conway et al. (2020). In other words, Arg1+ monocytes were recruited in the early stage of injury, whereas Mmp12+ macrophages participated in the whole process of renal repair. The cells had two different fates from node 2 onwards (Figure 4B), showing four completely different gene expression patterns (Figure 4D). We further performed a functional enrichment analysis of the DEGs (Figure 4E). Various macrophage subtypes showed anti-inflammatory activity, including defense response to the virus, positive regulation of chemokine ligand 2 production, cellular response to interleukin-4, and granulocyte chemotaxis. However, groups 3 and 4 were also involved in regulating cell adhesion and ECM organization and were believed to be highly associated with fibrosis.

### 3.4 Global alterations of cell-cell interactions between macrophages and other cell types

**FIGURE 5:**
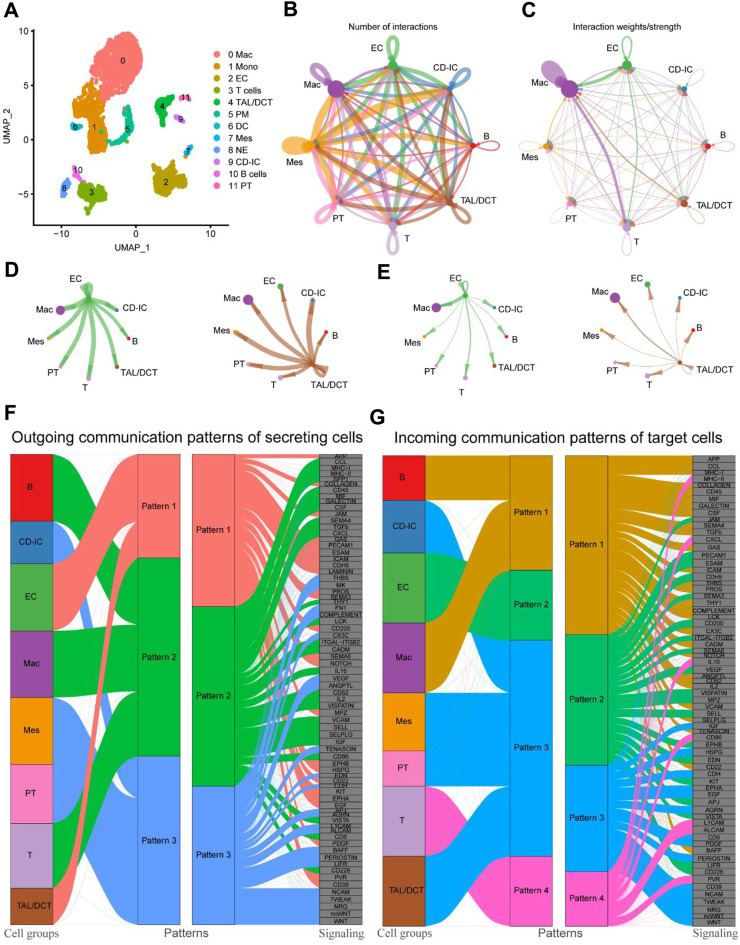
Cell-cell communications between cell subtypes in UUO7. **(A)** UMAP plot of UUO-7. Circle plots of the number of interactions **(B)** and interaction weights/strength **(C)**. Arrow indicated direction. Edge thickness indicated the strength of the relationship. The loops indicated autocrine circuits. The number of interactions **(D)** and interaction weights/strength **(E)** between EC or TAL/DCT cells and other cell types were displayed separately. **(F)** Outgoing communication patterns of secreting cells. **(G)** Incoming communication patterns of target cells. EC, endothelial cells; TAL, thick ascending limb; DCT, distal convoluted tubule.

Our results and previous reports suggest that macrophages may be deeply involved in fibrosis during kidney injury and repair (Conway et al., 2020; Wen et al., 2021). However, there are few reports on how macrophages transform during the whole process and which cells or cytokines give macrophages this “transformation signal.” The “CellChat” R package is a recently developed and effective tool for analyzing, inferencing, and visualizing cell-cell communication using the scRNA-seq database. In this study, the proportion of macrophages increased 51-fold, from 0.63% at the beginning to 32.26% at UUO7, and decreased to 9.79% after RUUO (Supplementary Table S1). Macrophages were hypothesized to play an essential role during this period. Thus, we re-clustered the UUO7 datasets using simplified global clustering for cell-cell interaction analysis. Twelve large subpopulations were identified (Figure 5A). The top five most highly expressed genes are shown in Supplementary Figure S3 to justify the rationality of clustering. The relationship between macrophages and other myeloid cell subtypes is relatively well-defined; therefore, we focused on the interaction between macrophages and other non-myeloid cells. The aggregated cell-cell communication networks are shown in Figures 5B, C, illuminating the number of interactions and the weight and strength with significant changes. Besides myeloid immune cells, macrophages were closely related to EC and TAL/DCT (Figures 5D, E). Multiple cell populations function together in certain communication patterns. There were three outgoing communication patterns of the secreting cells and four incoming patterns of the target cells (Figures 5F, G).

**FIGURE 6:**
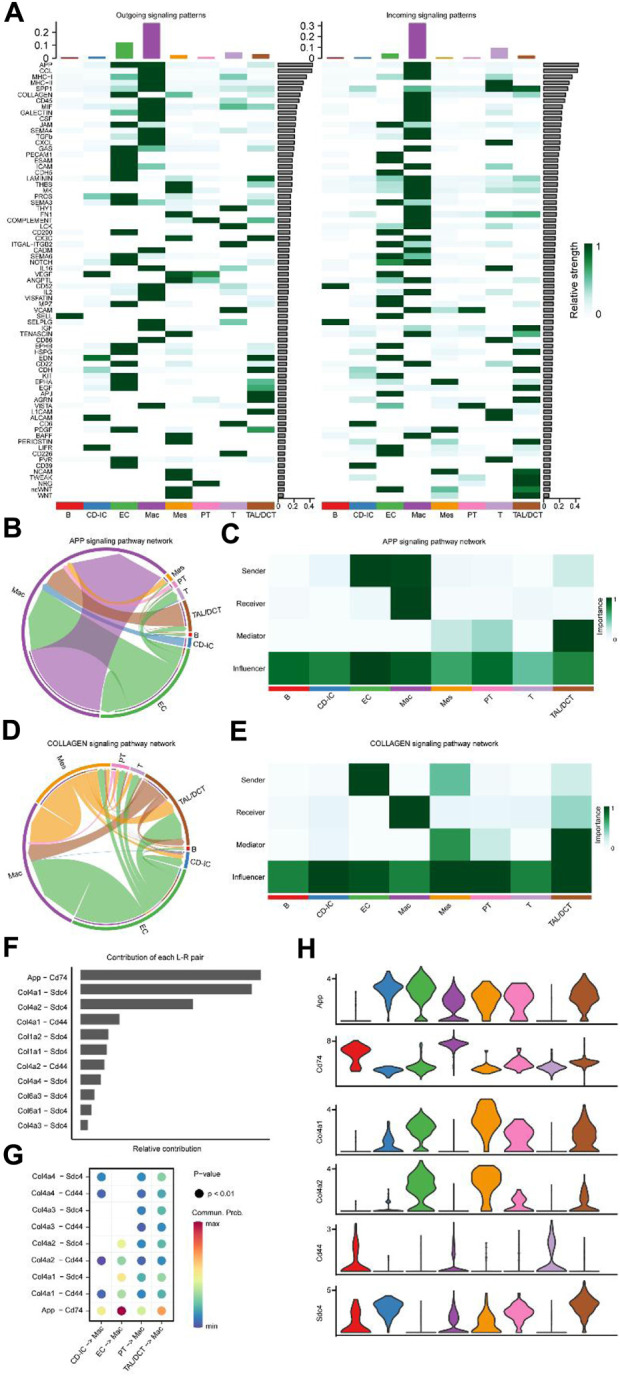
Major signaling changes and ligand-receptor pairs between cell subtypes of UUO7. **(A)** The relative strength of outgoing (Left) and ingoing (Right) signaling patterns for each cell subtype. Inferred APP **(B)** and COLLAGEN **(D)** signaling pathway network of cell subtypes. Heatmap of the APP **(C)** and COLLAGEN **(E)** signaling pathway network displaying relative importance of each cell subtype ranked according to computed four network centrality measures. **(F)** The bar plot displayed each ligand-receptor pair’s relative contribution toward the APP and COLLAGEN signaling pathways. **(G)** Ligand-receptor pairs mediated cellular communication between other cell types and macrophages. The dot color represented the values of communication probability, and the size was in proportion to the *P* value. **(H)** Violin plots show the expression of representative genes in each cell type.

Heat maps of outgoing (left panel) and incoming signaling patterns (right panel) were generated (Figure 6A) to identify intercellular communication changes among cell types in detail. Seventy-three critical signaling pathways were identified. With other types of cells as outgoing signaling and macrophages as incoming signaling, amyloid beta precursor protein (APP) and COLLAGEN signaling patterns showed outstanding performances. The effect intensity of APP (Figures 6B, C) and COLLAGEN (Figures 6D, E) signaling pattern networks among different cell types is shown in detail; the EC acts as a sender in two signaling patterns. Macrophages are the primary receivers; both cell types are important influencers. Notably, the contribution of various L-R pairs showed that the most significant difference between ECs and macrophages was in APP-CD74 (Figures 6F, G). In addition, the effect of EC on macrophages was shown in the L-R pairs of Col4a1-Sdc4 and APP-CD74, confirmed by the specific gene expression violin diagram (Figure 6H).

### 3.5 The L-R pair of APP-CD74 mediates the function of macrophages in renal injury

**FIGURE 7:**
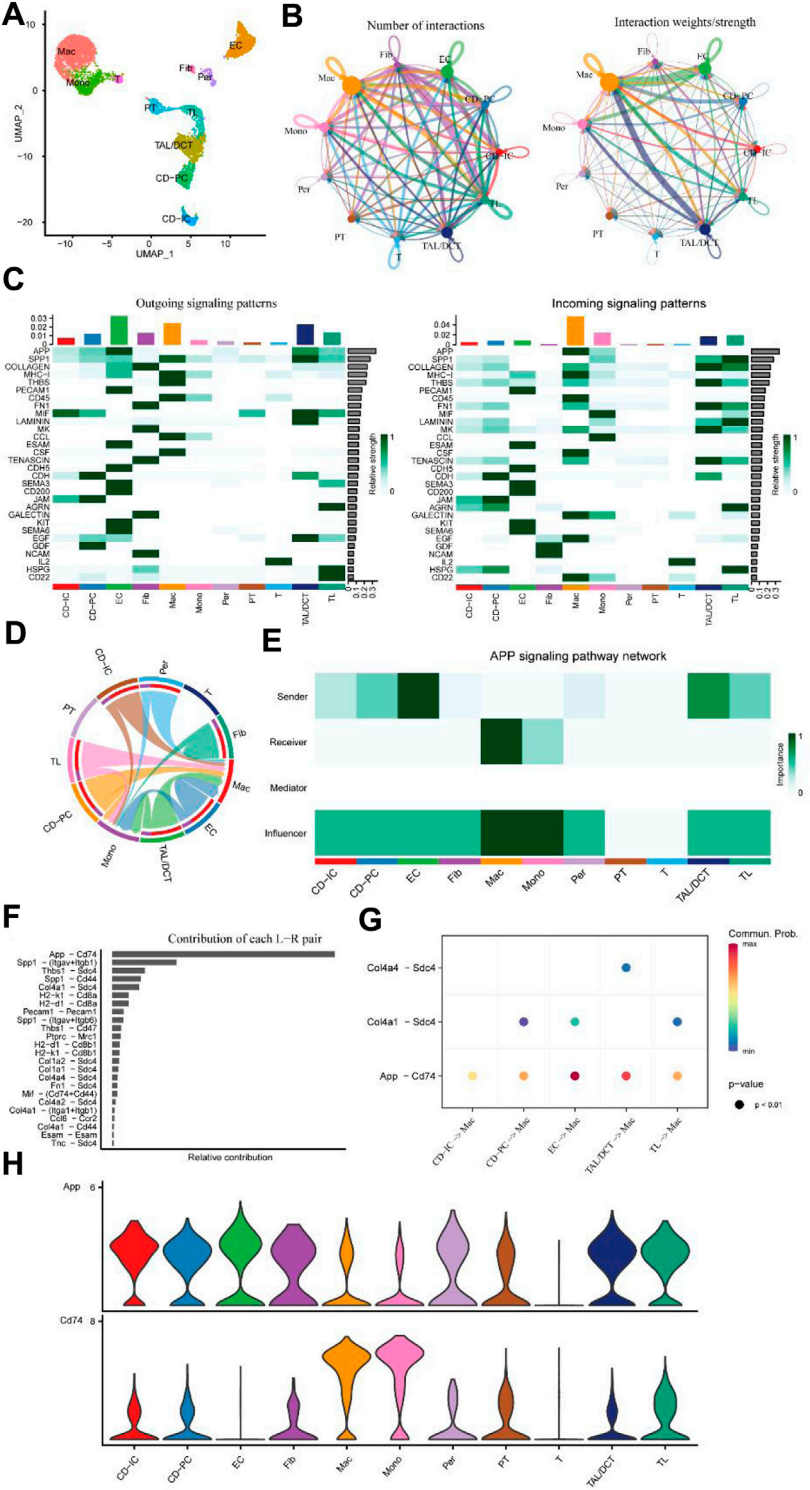
Independent external scRNA-seq dataset for mouse IRI (GSE161201). **(A)** UMAP plot of IRI 7. **(B)** Circle plots of the number of interactions and interaction weights/strength. Arrow indicated direction. Edge thickness indicated the strength of the relationship. The loops indicated autocrine circuits. **(C)** Outgoing and incoming signaling patterns of cells. **(D)** Inferred APP signaling pathway network of cell subtypes. **(E)** Heatmap of the APP signaling pathway network displaying the relative importance of each cell subtype ranked according to computed four network centrality measures. **(F)** The bar plot displays each ligand-receptor pair’s relative contribution toward the APP signaling pathways. **(G)** Ligand-receptor pairs mediated cellular communication between other cell types and macrophages. The dot color represented the values of communication probability, and the size was in proportion to the *P* value. **(H)** Violin plots show the expression of representative genes in each cell type.

To validate the two L-R pairs between ECs and macrophages identified above in the UUO kidney injury model, we used an independent external scRNA-seq dataset (GSE161201) of IRI in mice. Using this dataset, Shintaro et al. obtained kidneys for Drop-seq at 6 h, 1, 7, and 21 days after IRI (Ide et al., 2021). Consistent with the above, we obtained sequencing data on day 7 for further analysis. After using the Seurat algorithm for standardization, we obtained 19,841 transcriptome data points from 6,564 cells. Consistent with Shintaro et al., we performed unsupervised clustering of all kidney cells on day 7; 11 cell clusters were classified (Figure 7A; Supplementary Figures S4A, B; Supplementary Spreadsheet S3). It was not difficult to find that the number of macrophages and EC occupied a considerable proportion on the 7th day of IRI. At this point, the number, weight, and strength of various cellular interactions were visualized (Figure 7B). The weight/strength of the interaction between EC and macrophages and the relationship between TAL/DCT and macrophages were particularly significant. Further detailed analysis of intercellular outgoing and incoming signaling patterns revealed that the APP and COLLAGEN signal strengths ranked highest (Figure 7C). The APP signaling pattern also showed a strong correlation between EC and macrophages (Figures 7D–H), particularly the L-R pair of APP-CD74. In contrast to the UUO model data findings of Conway et al. (2020), the COLLAGEN signal in the IRI model was predominantly enriched in fibroblasts and TAL/DCT, followed by EC and macrophages (Figure 7C; Supplementary Figures S5A–C). Thus, we proposed that the L-R pair of APP-CD74 played a crucial role in endothelial-macrophage intercellular communication during kidney injury.

**FIGURE 8:**
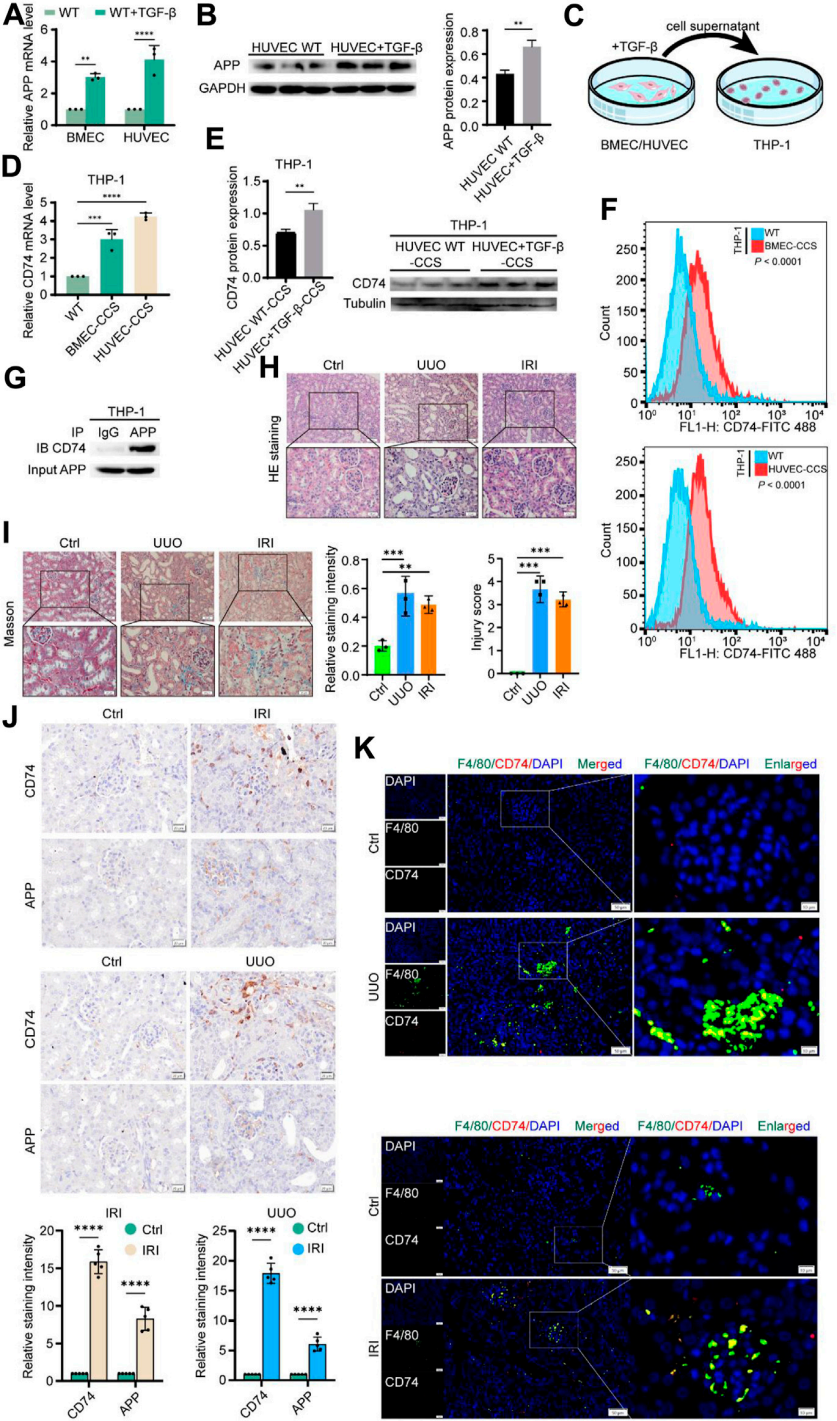
Experimental validation of APP-CD74 interaction between endothelial cells and macrophages. **(A)** APP mRNA levels after TGF-β treatment of BMEC and HUVEC. **(B)** Western blot results of APP protein expression and its statistical graph after TGF-β treatment of HUVEC. **(C)** Pattern diagram of co-culturing TGF - β treated BMEC or HUVEC culture medium supernatant with THP-1 cells. **(D)** CD74 mRNA levels in macrophages after co-culture. **(E)** Western blot results of CD74 protein expression and its statistical graph in macrophages after co-culture. **(F)** Flow cytometric results of CD74-positive macrophages after co-culture. **(G)** Co-IP indicates that the APP was directly integrated with CD74. **(H)** HE staining and injury score confirmed the successful construction of UUO and IRI models. **(I)** Masson staining and statistical graph confirm the successful construction of UUO and IRI models. **(J)** Representative images of immunohistochemical staining and its statistical graph of APP and CD74 in mouse IRI and UUO models. **(K)** Representative images of immunohistochemical staining for co-localization of F4/80 and CD74 in mouse IRI and UUO models. BMEC, brain microvascular endothelial cells; HUVES, human umbilical vein endothelial cells; CCS, co-culture system.

We further validated these results at the *in vivo* and *in vitro* levels, respectively. First, we treated BMEC and HUVEC cells with or without TGF-β 10 ng/mL for 24 h. The qPCR results suggested that TGF-β treatment significantly increased APP levels in endothelial cells (Figure 8A). Western blot results also demonstrated this at the protein level (Figure 8B). Afterwards, we added the supernatant of TGF-β-treated endothelial cells to the PMA-induced THP-1 cells and continued the culture. After 24 h THP-1 cells were collected for subsequent experiments (Figure 8C). Similarly, we detected an upregulation of CD74 expression in macrophages at both mRNA and protein levels (Figures 8D, E). Flow cytometry analysis also confirmed a significant increase in the number of CD74 positive macrophages (Figure 8F). Co-IP results confirm that APP and CD74 have a direct interaction (Figure 8G). To reflect whether this endothelial-macrophage communication process also exists in the real *in vivo* environment, we performed UUO and IRI modeling in mice. HE staining, tissue damage score, and Masson’s results all confirmed the successful construction of the model (Figures 8H, I). UUO kidneys show a marked irregular arrangement of renal tubules and dilatation of the tubular lumen. The tubular epithelial cells of IRI kidneys appear swollen with hydropic or vacuolar degeneration and loss of cellular brush border. Immunohistochemical results showed a significant increase in APP and CD74 deposition in the injured kidneys (Figure 8J). Tissue immunofluorescence observed a large number of macrophage infiltrates (green) all around the glomeruli and co-localized with CD74 (red, merged in yellow) (Figure 8K). The positive cells were mainly distributed in the tubular cells, tubulointerstitial area, and glomeruli. Thus, we have indeed confirmed that during renal injury, endothelial cells are able to express APP and transmit this signal to macrophages via CD74, which in turn causes a subsequent series of changes in the injury and anti-injury processes.

## 4 Discussion

With the advent of scRNA-seq technology, it is possible to simultaneously study the number and transcription dynamics of cells during the occurrence of diseases and model the development trajectory and interactions (Luecken and Theis, 2019; Jin et al., 2021). Conway et al. (2020) simulated the process of kidney damage and repair by establishing mouse UUO and RUUO models and conducted a scRNA-seq analysis of the renal cortex at different time points. Their study revealed heterogeneity of myeloid cells during this process. Arg1+ pro-inflammatory monocytes are recruited during the early stages of injury. Ccr2+ macrophages accumulate in the late stage of injury, while Mmp12+ macrophages may be related to the repair of kidney injury. In this study, we downloaded the sequencing dataset by Conway et al. (2020) (GSE140023) and performed a reanalysis. In addition, we focused on macrophages during injury and repair and investigated whether they are transformed because they receive regulation from other non-immune cells.

Macrophages are innate immune cells involved in acute kidney injury (AKI), repair, and fibrosis, playing an important role in body defense and tissue homeostasis (Huen and Cantley, 2017; Allison, 2018). Macrophages are recruited to trigger an early inflammatory response in the kidney after infection, ischemia-reperfusion, nephrotoxic drugs, and other injuries; this is one of the manifestations of the body in actively fighting against external injuries. However, prolonged inflammation and improper repair can lead to permanent structural and functional changes, which are the primary causes of the progression of AKI to CKD. In the present study, the number of macrophages changed unexpectedly. In addition to well-known functions, such as activation of innate immune responses, neutrophil chemotaxis, and B cell or Toll-like receptor signaling pathways, GO and KEGG functional enrichment analyses showed that macrophages played an active role in osteoclast differentiation and proteasomal pathways (Figure 2). However, the relationship between osteoclasts and fibrosis remains unclear. Furthermore, fibroblast growth factor 2 can inhibit osteoclast differentiation by counteracting macrophage colony-stimulating factor (Chikazu et al., 2001). In addition, periodontal fibroblasts can induce osteoclast differentiation and maturation (Bloemen et al., 2010). The protective role of the proteasome pathway in the fibrosis of the skin (Shen et al., 2021), lung (Inui et al., 2021), and kidneys (Zeniya et al., 2017) has been widely reported. Therefore, macrophages may affect renal fibrosis through osteoclast differentiation and the proteasomal pathway.

Monocyte differentiation is a major source of macrophages that varies unexpectedly. Therefore, studying the dynamic changes in monocytes and macrophages in different subtypes is necessary. This is facilitated by pseudo-time trajectory analysis of scRNA-seq (Abbas et al., 2020). Ly6c+ monocytes are progenitors of inflammatory macrophages (Shi and Pamer, 2011), consistent with our results (Figure 4). Along the pseudo-time trajectory, Ly6c2+ monocytes gradually differentiate into Mmp12+, Pcna+, AP, and ISGhi macrophages. Mmp12, also called macrophage elastase, is mainly derived from macrophages; its main function is to degrade elastin (Chou et al., 2016), participate in inflammation (Nenan et al., 2005), and ECM remodeling (England et al., 2011). MMP12 promotes phagocytosis by cleaving C3b and iC3b opsonized particles, while inactivating C3a and C5a to downregulate inflammatory cell infiltration and resolve inflammation to prevent ongoing tissue damage (Bellac et al., 2014). Tang et al. (2020) showed that cytoplasmic PCNA is involved in osteoclast differentiation in osteoporosis. In addition, the kidneys of high-phosphate diet mice showed higher PCNA expression, macrophage infiltration, and fibrosis (Duayer et al., 2021). Further functional enrichment analysis revealed that these cells with different differentiation directions had specific functions in regulating cell adhesion and organization of the ECM (Figures 4D, E). We hypothesized that other cells may be connected to these “advanced” macrophage types, participating in macrophage phagocytosis and the ECM remodeling process, thereby affecting renal fibrosis.

The effects of macrophages on EC under various pathological conditions have been previously reported. Uremic serum treatment results in increased migration and adhesion of EC by macrophages (Trojanowicz et al., 2014). Macrophages play a direct role in the migration of EC and the formation of neovascularization in hypoxic nerve tissues (Ren et al., 2018). The TGF-β/Smad signaling pathway is the core of renal fibrosis. Macrophages and tubular epithelial cells are important sources of TGF-β changes in UUO renal fibrosis model (Cui et al., 2014). Macrophage-derived Wnt7b is critical for renal tubular epithelial regeneration during renal regeneration (Lin et al., 2010). On the other hand, the impact of endothelial/epithelial cells on macrophages mainly involves the recruitment of macrophages by endothelial cells and the induction of macrophage polarization towards M1 or M2 by renal tubular epithelial cells at different stages of AKI, thereby influencing their functional roles (Deng et al., 2022).

However, the precise mechanisms and signaling pathways through which endothelial/epithelial cells influence macrophage behavior and function are not yet fully clear. In this study, we observed a strong effect of EC and TAL/DCT on macrophages regarding the number and weight/intensity in UUO and IRI models. APP-CD74 had the highest relative contribution from the ligand-receptor pairs. In addition, the Col4a1-Sdc4 pair played a partial role. Col4a1 encodes type IV collagen, one of the main components of the ECM, and its role in renal fibrosis is self-evident. Sdc4 is a potential partner of transglutaminase-2 in pulmonary (Tanino et al., 2019) and cardiac (Herum et al., 2020) fibrosis. Sdc4 knockout results in decreased extracellular transglutaminase-2 levels and prevents tubulointerstitial fibrosis (Scarpellini et al., 2014; Wee et al., 2019). Based on the scRNA-seq data validated in two different kidney injury models, ECs may mediate macrophage function to promote fibrosis through the APP-CD74 pathway screened in this study.

APP was first discovered in the context of Alzheimer’s disease, and its function has been gradually elucidated over the years. It is associated with cell adhesion (Young-Pearse et al., 2008), fibroblast proliferation (Saitoh et al., 1989), and neuronal stem cell differentiation (Caille et al., 2004). The APP molecular structure can bind to an ECM component, potentially providing insight into its possible profibrogenic function (Dawkins and Small, 2014). Kainic acid lesions of the rat striatum caused an increase in APP immunoreactivity in neurons and neurites, some of which were subsequently phagocytosed by reactive microglia/macrophages (Shigematsu et al., 1992). Furthermore, platelet-derived APP in atherosclerosis is processed by protein hydrolysis to Abeta, which promotes inducible nitric oxide synthase expression and thus induces macrophage activation (De Meyer et al., 2002). CD74 is a non-polymorphic type II transmembrane glycoprotein with various biological functions in physiological and pathological states. CD74 plays an important role in many inflammatory diseases such as skin fibrosis (Borrelli et al., 2020) and Alzheimer’s disease (Bryan et al., 2008). CD74 regulates T and B cell development, dendritic cell motility, macrophage inflammation and thymic selection. Activation of the receptor complex CD74/CD44 leads to the activation of multiple intracellular signaling pathways, such as extracellular signal-regulated kinases 1 and 2, the PI3K-Akt signaling cascade, NF-κB and the AMP-activated protein kinase pathway (Su et al., 2017). The macrophage migration inhibitory factor-CD74/CD44 axis is upregulated in glomerulocytes and is closely associated with the pathological proliferation (Djudjaj et al., 2016).

The intense APP-CD74 communication signal between endothelial cells and macrophages in both kidney injury models (UUO and IRI) gives us a sufficient reason to hypothesize that endothelial cells may impact macrophage function via the APP-CD74 axis, thereby influencing the fibrotic process during kidney injury and repair, further experimental verification for which was carried out. TGF-β is a well-known pro-fibrotic factor in kidney disease and is often used as an induction model to study the regulatory mechanisms of fibrotic disease *in vitro*. We observed elevated APP expression in both TGF-β-treated BMEC and HUVEC endothelial cell lines. In both UUO and IRI kidneys, a considerable deposition of APP was observed. These results suggest that endothelial cells show a positive response during kidney injury. Furthermore, we added the above endothelial cell culture supernatants to macrophages for co-culture. The PMA-induced THP-1 cells also showed a significant upregulation of CD74 expression. Notably, this was confirmed by immunohistochemical and immunofluorescence results from IRI and UUO kidneys. Overall, these results completely support our previous speculation.

Nevertheless, there are some limitations to this study. Compared with single-nucleus RNA-seq, the scRNA-seq technique applied in our two selected kidney injury models obtained more immune cells (Slyper et al., 2020). However, the macrophage counts at time points other than UUO7 are still insufficient to support our dynamic monitoring of the changes in communication between endothelial cells and macrophages. Further improvements should ideally include conducting additional sample assays to improve yields and performing further experiments such as target gene knockouts to validate our findings.

## 5 Conclusion

In this study, we confirmed the heterogeneity and pseudo-time trajectory of macrophages during renal injury and repair by in-depth mining of scRNA-seq profiles of the mouse UUO model. In addition, abundant cellular communication was revealed between endothelial cells and macrophages during renal injury in both UUO and IRI models; it was found that APP-CD74 plays a crucial function in this process. Furthermore, we validated these results in in vitro and *in vivo* experiments. Our findings suggest that the APP-CD74 pathway between endothelial cells and macrophages may affect kidney injury repair and is a potential therapeutic target for antifibrosis. In summary, these findings provide a valuable direction for understanding the molecular mechanisms of macrophage involvement in renal fibrosis, contributing to the delay in CKD.

## Figures and Tables

**Table:** 

**AKI**	Acute kidney injury
**AP**	Antigen presentation
**APP**	Amyloid beta precursor protein
**CD-ICs**	Collecting duct intercalated cells
**CD-PCs**	Collecting duct principal cells
**CKD**	Chronic kidney disease
**DCs**	Dendritic cells
**DEGs**	Differentially-expressed genes
**ECs**	Endothelial cells
**ECM**	Extracellular matrix
**EMT**	Epithelial-mesenchymal transformation
**GEO**	Gene expression omnibus
**GO**	Gene ontology
**ISGhi**	Interferon-stimulated gene high expression
**IRI**	Ischemia-reperfusion injury
**KEGG**	Kyoto encyclopedia of genes and genomes
**L-R**	Ligand-receptor
**Mes**	Mesangial cells
**Monos**	Monocytes
**NEs**	Neutrophils
**Pers**	Pericytes
**PECs**	Parietal epithelial cells
**PMs**	Proliferating monocytes
**Pcna**	Proliferating cell nuclear antigen
**PT**	Proximal tubule
**Pods**	Podocytes
**RUUO**	Reversible unilateral ureteral obstruction
**scRNA-seq**	Single-cell RNA sequencing
**TAL/DCTs**	Thick ascending limb/distal convoluted tubules
**TLs**	Thin limb cells
**UUO**	Unilateral ureteral obstruction
**UMAP**	Uniform manifold approximation and projection

## Data Availability

The original contributions presented in the study are included in the article/Supplementary Material, further inquiries can be directed to the corresponding authors.
