# Supplementary material for: APP-CD74 axis mediates endothelial cell-macrophage communication to promote kidney injury and fibrosis
Source: Front Pharmacol. 2024 Sep 16;15:1437113. doi: 10.3389/fphar.2024.1437113 (PMC11439715; doi:10.3389/fphar.2024.1437113)
Supplement: Supplementary file 3 [file Presentation1.pdf]

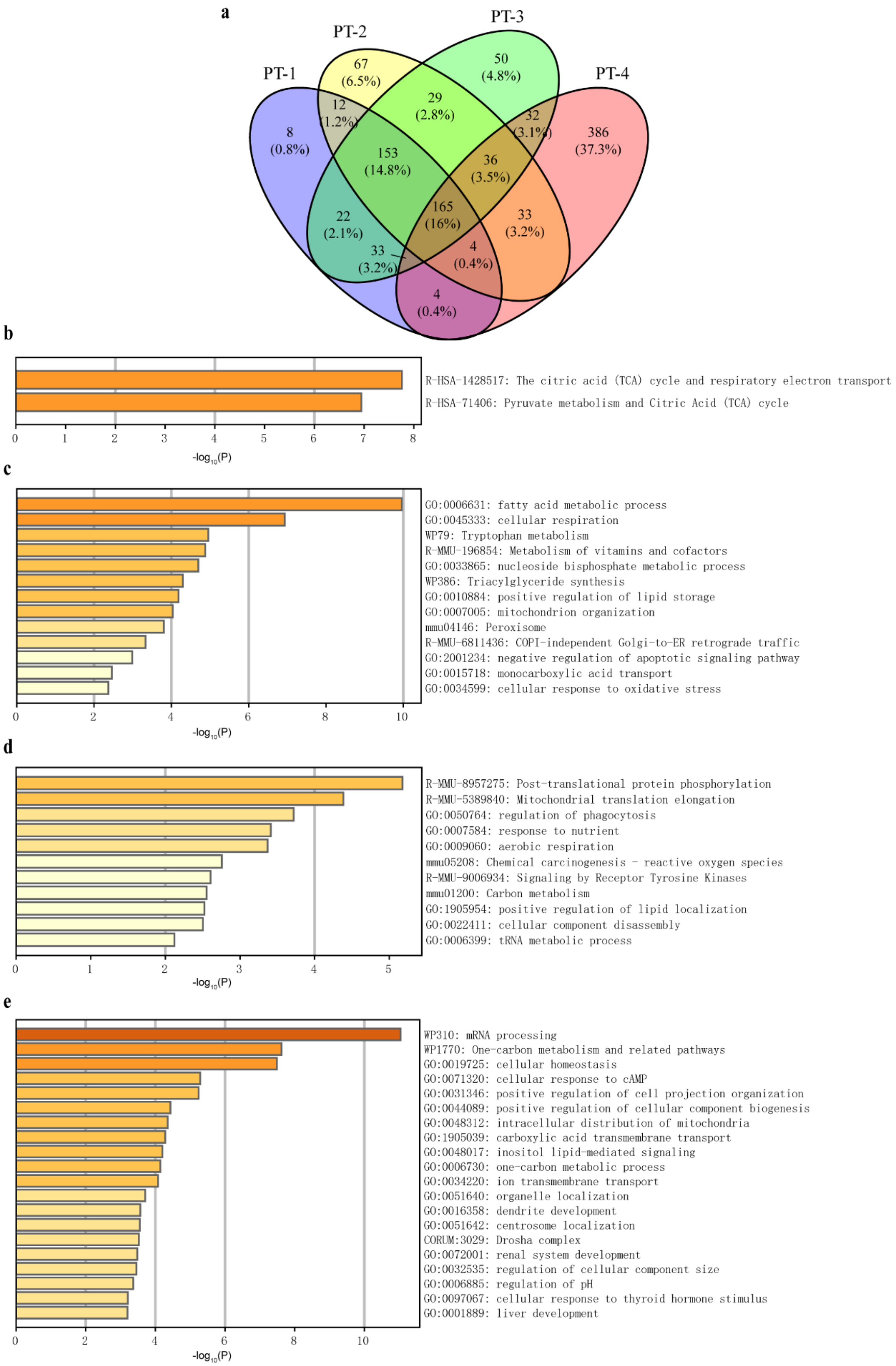

Supplementary Figure 1. Differential analysis of PT-1-4 cells. (**a**)The differentially expressed genes of PT-1-4. Functional enrichment analysis of differentially expressed genes of PT-1 (**b**), PT-2 (**c**), PT-3 (**d**), and PT-4 (**e**) compared with the other three groups were expressed as the -log10 (P) adjusted for multiple comparisons. PT, proximal tubule.

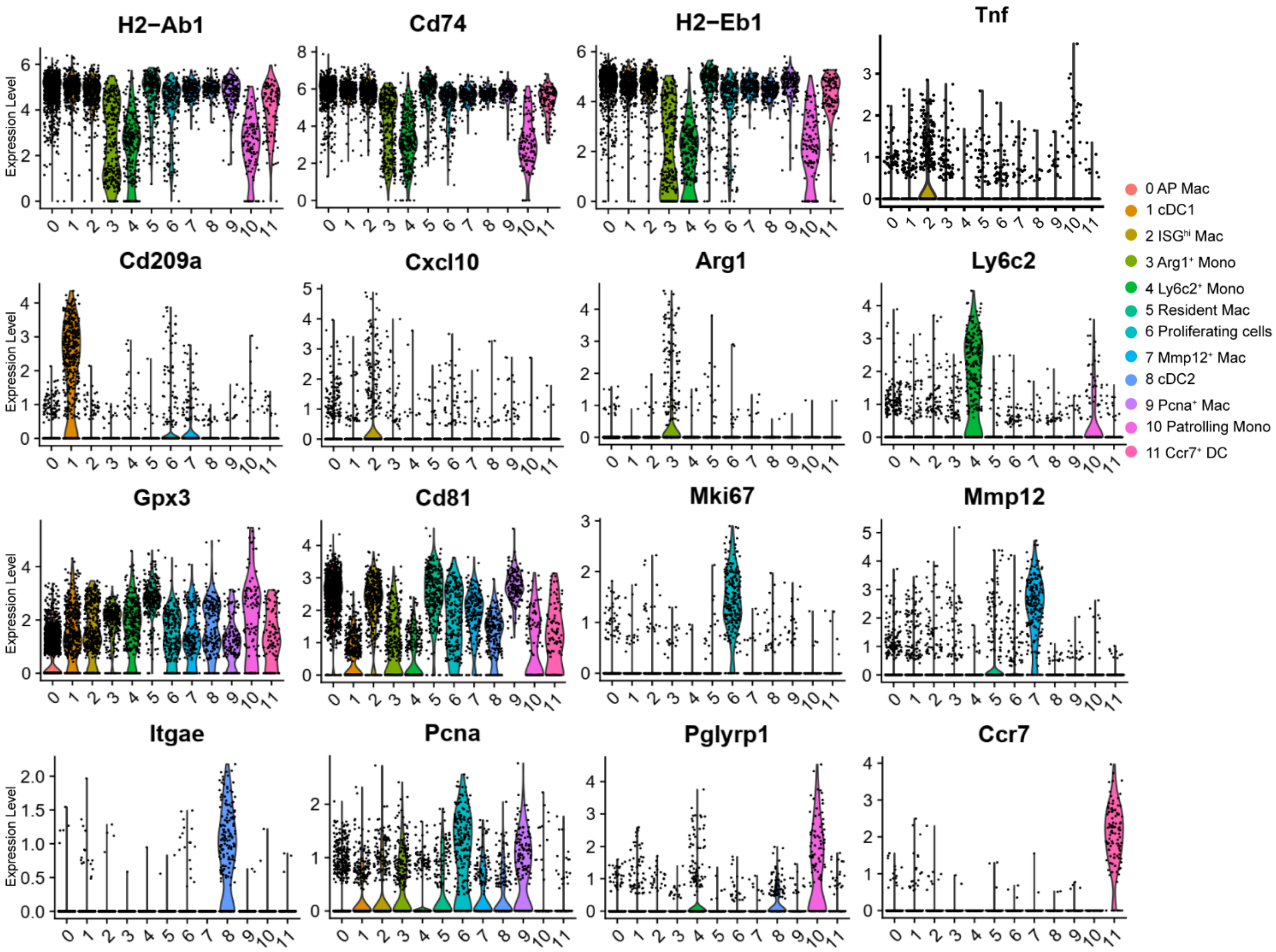

Supplementary Figure 2. Marker genes of myeloid cell subtypes.

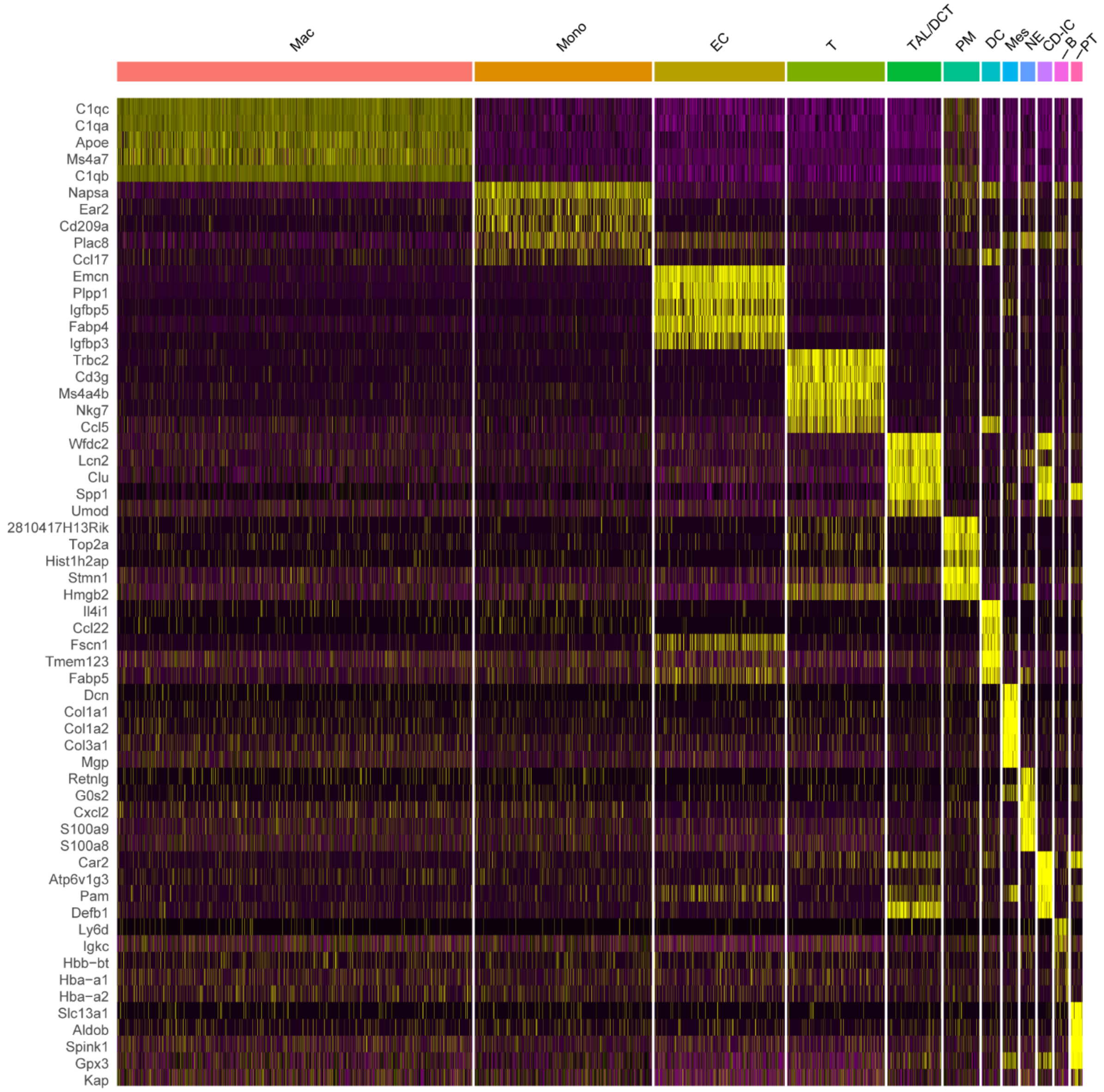

Supplementary Figure 3. Heat map displaying gene expression patterns of the top 5 in UUO7 each cluster.

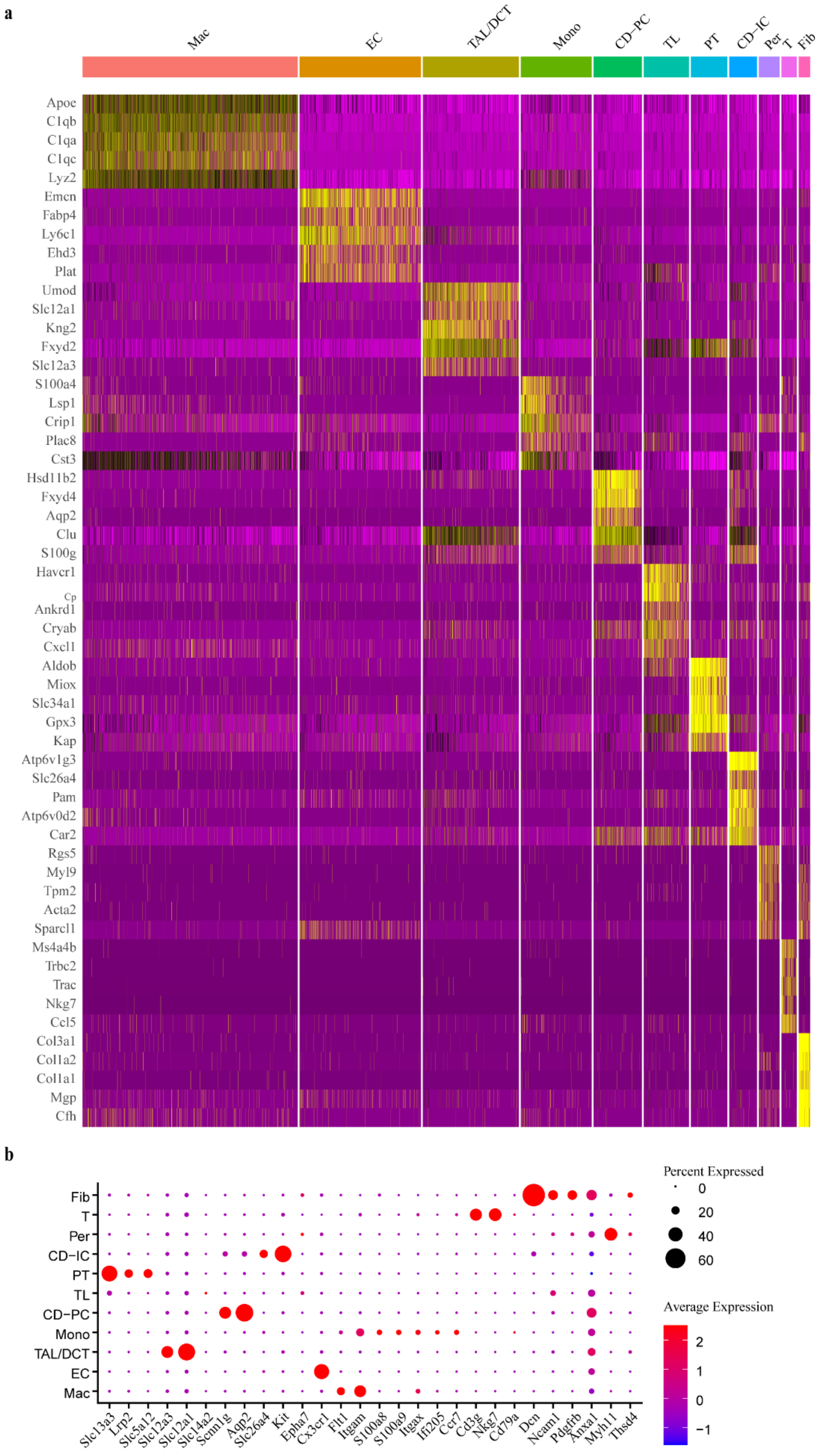

Supplementary Figure 4. Identified different cell subsets in the kidney of IRI 7th day mice from GSE161201. (**a**) Heat map displaying gene expression patterns of the top 5 in each cluster. (**b**) Dot plot showing differential expression levels of some well-known marker genes for cell clusters.

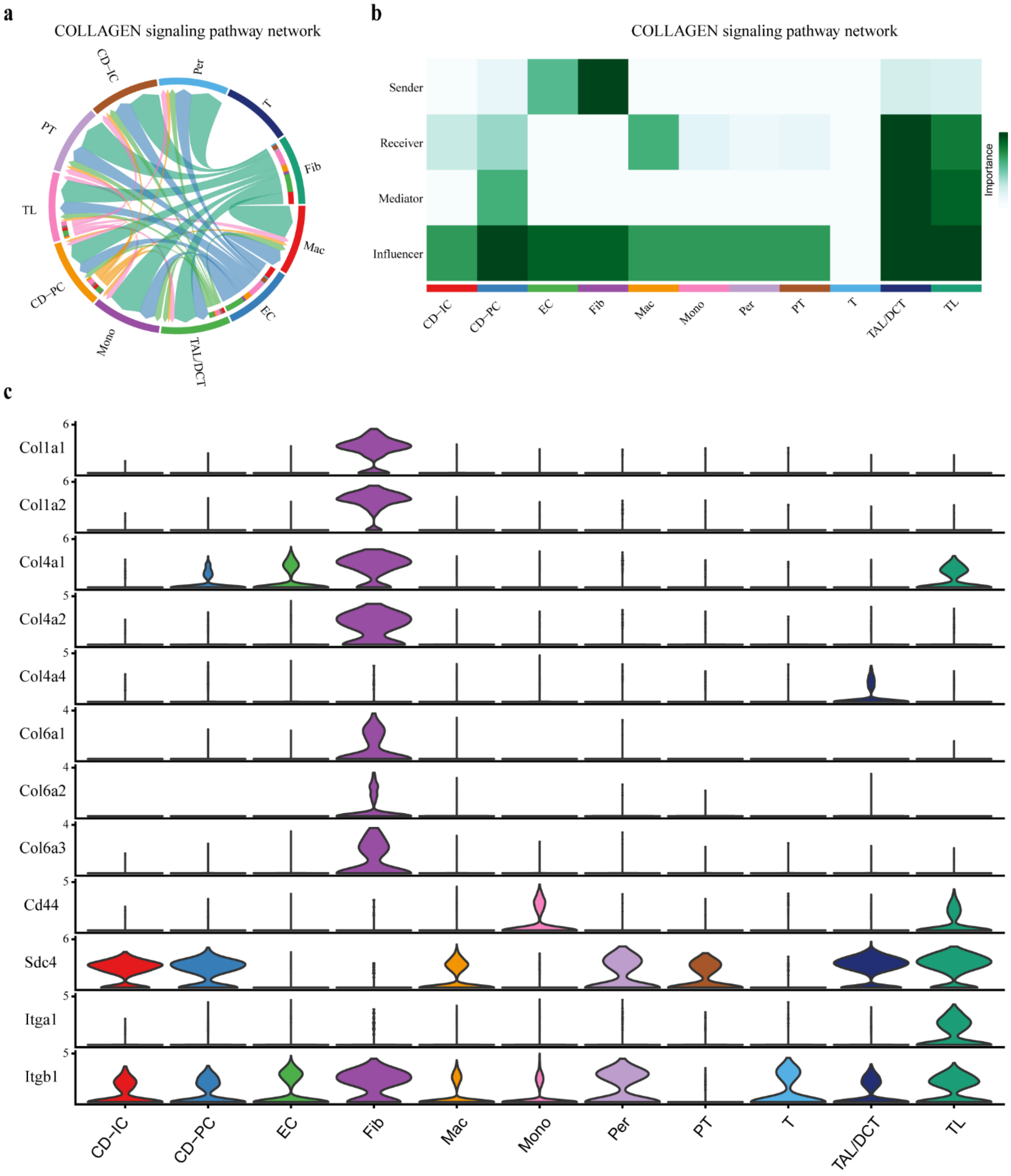

Supplementary Figure 5. COLLAGEN signaling pathway network. (**a**) Inferred COLLAGEN signaling pathway network of cell subtypes. (**b**) Heatmap of the COLLAGEN signaling pathway network displaying relative importance of each cell subtype ranked according to computed four network centrality measures. (**c**) Violin plots show the expression of representative genes in each cell type.

Supplementary Table 1. The percentage of all 22 cell types identified in different groups.

Cluster	Cell type	Number of cells				Percentage (%)			
		Sham	UUO2	UUO7	R-UUO	Sham	UUO2	UUO7	R-UUO
0	PT-1	2374	9	0	59	46.49	0.37	0	1.70
1	Mac	32	30	1439	341	0.63	1.22	32.26	9.79
2	PT-2	1300	4	58	267	25.46	0.16	1.30	7.67
3	PT-3	11	535	70	665	0.22	21.76	1.57	19.09
4	TAL/DCT	314	324	160	248	6.15	13.18	3.59	7.12
5	CD-PC	209	261	318	257	4.09	10.61	7.13	7.38
6	PT-4	417	161	146	296	8.17	6.55	3.27	8.50
7	T cell	27	142	395	281	0.53	5.78	8.86	8.07
8	EC-1	115	111	353	262	2.25	4.51	7.92	7.52
9	Mono-1/NE	16	296	269	85	0.31	12.04	6.03	2.44
10	Mono-2	18	87	411	128	0.35	3.54	9.22	3.68
11	CD-IC	59	157	153	119	1.16	6.39	3.43	3.42
12	EC-2	119	52	159	120	2.33	2.12	3.57	3.45
13	PM	0	124	133	22	0	5.04	2.98	0.63
14	DC-1	2	24	117	65	0.04	0.98	2.62	1.87
15	TL	51	66	45	34	1.00	2.69	1.01	0.98
16	Fib	0	4	83	65	0	0.16	1.86	1.87
17	Pod/PEC	5	13	12	90	0.10	0.53	0.27	2.59
18	DC-2	0	4	81	27	0	0.16	1.82	0.78
19	B cell	4	25	42	29	0.08	1.02	0.94	0.83
20	Mes	26	16	11	2	0.51	0.65	0.25	0.06
21	Per	7	14	5	21	0.14	0.57	0.11	0.60
